# Immunological evaluation of recombination PRRSV GP3 and GP5 DNA vaccines *in vivo*

**DOI:** 10.3389/fcimb.2022.1016897

**Published:** 2022-10-06

**Authors:** Guanyu Zhao, Jiaqi Zhang, Wenchao Sun, Changzhan Xie, He Zhang, Yan Gao, Shubo Wen, Zhuo Ha, Fulong Nan, Xiangyu Zhu, Sheng Feng, Xinyu Cao, Ying Zhang, Yanzhu Zhu, Ningyi Jin, Huijun Lu

**Affiliations:** ^1^ College of Veterinary Medicine, College of Animal Science, Jilin University, Changchun, China; ^2^ Changchun Veterinary Research Institute, Chinese Academy of Agricultural Sciences, Changchun, China; ^3^ Institute of Special Animal and Plant Sciences, Chinese Academy of Agricultural Sciences, Changchun, China; ^4^ Animal Science and Technology College, Jilin Agriculture Science and Technology University, Jilin, China

**Keywords:** PRRSV, GP3, GP5, immune response, DNA vaccine, pig

## Abstract

The porcine reproductive and respiratory syndrome virus (PRRSV) is a threat to the health of pigs worldwide, but commercially available vaccines offer limited protection against PRRSV infection. It is necessary to develop a more effective DNA vaccine. The immunological effects of DNA vaccines with three adjuvants were examined in pigs (*Susscrofa domestica*) challenged with PRRSV. These DNA vaccines, which encoded PRRSV GP3 and GP5, were formulated with A1, A2, and A3. Serum specific and neutralizing antibodies, IL-4, IFN-γ, IL-2, IL-10, CD4^+^ and CD8^+^T-lymphocytes, health status, histopathology, and viral loads were determined. The results showed that the use of adjuvant A3 led to higher levels of neutralizing antibodies and a lower viral load in pigs compared to the other adjuvants. The neutralizing antibody titers of the pVAX-GP35+A1 and pVAX-GP35+A3 groups reached a peak of 1:19 at 35 dpi. The maximum concentration of IL-4 was 136.77 pg/mL in the pVAX-GP35+A3 group. At 35 dpi, the IFN-γ concentration in the pVAX-GP35+A1 group was 227.4 pg/mL. pVAX-GP35+A3 group shows the highest IL-2 and IL-10 expression to the peak of 597.6 pg/mL and 189.1 pg/mL, respectively. We found a formulation demonstrated beneficial immune outcomes. This study provides an alternative vaccine to protect pigs from PRRSV.

## Introduction

For more than two decades, porcine reproductive and respiratory syndrome (PRRS) has been considered one of the most commercially important swine diseases worldwide. In the United States, PRRS is responsible for at least $6 million a year in losses to the pig industry in the United States (Neumann et al., 2005), besides, all over the world (Lunney et al., 2010). Because the hypoxia caused by respiratory distress turn the ears blue, PRRS was initially called blue-ear pig sickness (Wensvoort et al., 1991). In Southeast Asia, PRRS virus (PRRSV) infection was initially thought to be associated with hyperthermia, which manifested as severe respiratory disease and caused significant mortality in pigs of all ages (Tian et al., 2007). Since PRRSV has the ability to suppress the host’s immune system, it increases susceptibility to secondary infections and subsequently, more serious chronic diseases (Van Reeth et al., 1996). PRRSV modified live vaccines (MLVs), most widely used recently, are capable of providing moderate protection against homologous viruses and limited protection against other gene types of PRRSV (Kimman et al., 2009). Moreover, the use of MLV associated with a number of hazards. The virus may cause severe sickness in exposed pigs. The MLV inoculum may contain adventitious infectious agents that allow PRRSV to circulate throughout the herd and spread to other herds (Mengeling, 2005a; Mengeling, 2005b). Moreover, a risk of viral shedding after immunization with live attenuated vaccines has been found, leading to latent infection in healthy animals (Zhu et al., 2022). High safety is the characteristic of inactivated vaccine. Due to the weak cellular immune effect, many people have a difficult choice between an inactivated vaccine and MLV. Consequently, due to their superior safety profile, comprehensive humoral and cellular immune effects DNA vaccines have been approved globally (Renukaradhya et al., 2015). Avian influenza DNA vaccine was allowed to be listed in China (Jiang et al., 2007), DNA vaccine for SARS-CoV-2 was urgently approved for use in India (Mallapaty, 2021), which is the first time that DNA vaccine has been applied to humans.

DNA vaccines transfer genes that encode viral antigens *via* DNA vector plasmids. This strategy effectively actives humoral and cellular immune responses (Silveira et al., 2017). DNA vaccines have several advantages over traditional live or attenuated vaccines, including the ability to induce extensive cellular and humoral immune responses without the risk of viral replication, as well as being readily modifiable when changes to antigen encoding genes are necessary (Xu et al., 2014).

In the present study, pigs were immunized with a DNA vaccine in combination with various adjuvants, to assess its efficacy in stimulating a protective immune response. This study provides a practical, technical foundation for averting highly pathogenic PRRSV outbreaks in the future and reducing the economic losses associated with such outbreaks.

## Materials and methods

### Viruses, cells, and experimental animals

The highly pathogenic PRRSV GD strain was presented with the gift by South China Agriculture University. PRRSV was propagated in MARC-145 cells cultured in Dulbecco’s Modified Eagle Medium (DMEM, Gibco, Grand Island, NY, USA) supplemented with 10% fetal bovine serum (FBS, Gibco). Thirty-six Landrace-York crossbred piglets (weaned at 28 days old) were procured from a PRRSV-free farm in Changchun (Jilin, China). The pigs were detected by PCR and ELISA to confirm that PRRSV was seronegative. Pigs were separated into six groups (n = 6, pVAX vector for negative group, four vaccinazation groups, and inactived vaccine for positive group), and each group was housed separately.

### DNA vaccine and adjuvants

**Figure 1:**
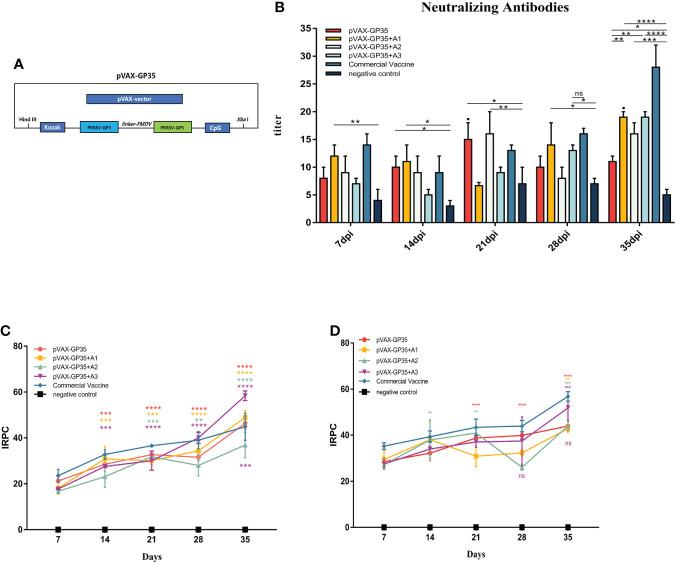
Changes of antibody levels after immunization. **(A)** DNA vaccine used in this study. **(B)** Neutralizing antibodies in pigs inoculated with the recombinant DNA vaccines. **(C)** IRPC of GP3 proteins through 35 dpi. All groups 14 days post-immunization (dpi) and beyond, except for the negative group, were positive. **(D)** IRPC of GP5 proteins through 35 dpi. All groups 7 dpi and beyond, except for the negative group, were positive. (*P < 0.05, **P < 0.01, ***P < 0.001, ****P < 0.0001). Mean ± SD of 3 data were shown in each group. The symbols above the fold line on the line graph denote current dpi against 7 dpi, with the colors denoting the immunized group. The colored symbols below the line represent the immunization group compared to the commercial vaccine. ns, no significant differences.

In a preliminary investigation, the DNA vaccine, pVAX-GP35, was developed (**Figure 1A**). Adjuvant A1 is a saponin extract, while adjuvant A2 is a water-in-oil-in-water mixture. A combination of adjuvants A1 and A2 make up adjuvant A3.

### Recombinant DNA vaccine groups and PRRSV challenge

Four groups, each containing six pigs, were immunized with the recombinant DNA vaccines (pVAX-GP35, pVAX-GP35+A1, pVAX-GP35+A2, or pVAX-GP35+A3). The empty pVAX vector was administered to the negative control group, while the commercial vaccine (Shanghai HILE BIO-TECHNOLOGY CO., LTD) was administered to the positive control group. Each pig in immunized groups were inoculated with 500 μg vaccine plasmids. Blood was collected from the pigs every seven days following inoculation, a total of 35 days. Second inoculation (booster) was given 21 days after the primary inoculation. All groups were challenged with 2×10^5^ TCID_50_ PRRSV at 35 days post immunization (dpi), and the viral load at 14 days post challenge was calculated.

### Neutralizing antibody detection

Serum samples collected from the pigs were heat-inactivated at 56°C for 0.5 hours. To detect neutralizing antibodies, 150 TCID_50_/mL of PRRSV was added to DMEM with 2% FBS, followed by successive 2-fold dilutions of the test sera (Fu et al., 2022), and incubated for one hour at 37°C. The mixture was then applied to monolayers of MARC-145 cells, which were cultured for four days at 37°C under 5% CO_2_. Using the Spearman-Karber method (Finney, 1985), the dilution of each serum sample providing a neutralizing antibody titer capable of protecting 50% of the cells against cytopathic effect (CPE) was calculated.

### Specific antibody detection

PRRSV GP3 and GP5 antibodies were detected according to the kit manufacturer’s instructions (Porcine PRRSV-GD GP3 Ab ELISA Kit ZR183, and Porcine PRRSV-GD GP5 Ab ELISA Kit ZR181, HCB, China). The IRPC was calculated using the OD_450_ values from the ELISA, using the following equation: IRPC=(OD_sample_ - OD_negative_) ÷ (OD_standard_ - OD_negative_)×100. IRPC values of more than 20 were classified as positive.

### Cytokine detection

Serum IL-4, IFN-γ, IL-2, and IL-10 analyses were performed ELISA kit (eBioscience, San Diego, CA, USA), following the manufacturer’s instructions.

### CD4^+^and CD8^+^T-lymphocyte analysis by flow cytometry

Peripheral blood lymphocytes (1×10^6^ cells in 100 ul) were extracted from the blood samples and treated with anti-pig CD3^+^, anti-pig CD8^+^, and anti-pig CD4^+^ antibodies for staining (BD Biosciences, SanDiego, USA). Flow cytometry was utilized to examine the results.

### Monitoring pig survival after PRRSV challenge

#### Health status

Five pigs were chosen at random to have their rectal temperature measured each day simultaneously, with the thermometer in place for five minutes. Rectal temperature was employed as a metric to assess illness progression following PRRSV infection. In addition, health after the PRRSV challenge was measured using an Activities of Daily Living (ADL) assessment (Romero-Ayuso et al., 2021). Appetite was assessed by feed intake per meal; mental status was assessed by responses to stimuli; head skin color was observed; breathing was judged by coughing and panting sounds. On a scale of 0-3, 0 represents severe symptoms, 1 represents moderate symptoms, 2 represents mild symptoms, and 3 represents no obvious symptoms.

#### Histopathological analysis

At 14 days post challenge, fresh lung tissue was harvested. Tissue sections were fixed, washed in water, dehydrated, embedded in wax, sectioned and heated, stained with hematoxylin and eosin (H&E), and pathologically examined under a microscope (200×).

#### Measurement of tissue viral loads in pigs

Three pigs were randomly selected from each group. Tissue specimens were obtained 14 days post challenge from the lungs, submandibular lymph nodes, and inguinal lymph nodes to quantify viral loads. RNA extractions were performed using an RNA extraction kit (Sangon Biotech Shanghai China), following manufacturer’s instructions. One-step RT-qPCR (ABI 7500 system, ThermoFisher, MA, USA) was performed on PRRSV ORF7 gene amplification to establish a quantitative fluorescence detection method (absolute quantification) which detects PRRSV viral load in tissues. Primers were designed in reference to the sequence of the PRRSV ORF7 gene generating the primers pair 5’-ATGGCCAGCCAGTCAATCA-3’ and 5’-TCGCCCTAATTGAATAGGTG-3’ (95°C 5min;95°C 30s, 53°C 30s, 72°C 30s, 35 circles; 72°C 5min).

#### Data analysis

GraphPad Prism (Version 5.0) was used to analyze the data, which was presented as the mean plus S.D. The Student’s *t* test was used in this study for statistical analysis. A *P* value of <0.05 was used to define statistically significant differences.

## Results

### Detection of neutralizing antibodies and GP3/GP5 antibodies in sera

Neutralizing antibody titers in pig sera were evaluated every seven days (**Figure 1B**). The neutralizing antibody titers of the pVAX-GP35 and pVAX-GP35+A2 groups were 1:15 and 1:16, respectively, after 21 dpi, which were greater than that of the negative control group (NC group) (P<0.05). The neutralizing antibody titers of the pVAX-GP35+A1 and pVAX-GP35+A3 groups reached a peak of 1:19 at 35 dpi, which were higher than those of the NC (P<0.01) and pVAX-GP35 groups (P<0.01). These findings reveal that pVAX-GP35+A1, pVAX-GP35+A2, and pVAX-GP35+A3 all promoted the development of neutralizing antibodies in pigs.

Specific antibodies targeting the PRRSV GP3 and GP5 proteins were found to have been induced by the recombinant DNA vaccinations. GP3 antibody levels were found to have increased after 35 dpi (**Figure 1C**). After 7 dpi, only the pVAX-GP35 and commercial inactivated vaccine groups had developed an antibody response to GP3. The GP3 antibody levels in the pVAX-GP35+A3 group increased substantially at 21 dpi. The IRPC of the pVAX-GP35 group was 46.36, the IRPC of the pVAX-GP35+A1 group was 48.93, and the IRPC of the pVAX-GP35+A3 group was 58.36 (P<0.05) at 35 dpi. Antibodies to GP5 began to appear at 7 dpi, and the data demonstrated an overall rising trend within 35 dpi (**Figure 1D**). At 28 dpi, the GP5 antibody levels in the pVAX-GP35+A2 group fluctuated. At 35 dpi, the IRPC reached its most remarkable point in each group, with all adjuvant groups outperforming the pVAX-GP35 group. All vaccine groups were slightly less effective than the commercially available inactivated vaccine (P>0.05).

### Serum cytokine analysis and T-lymphocyte subtyping

**Figure 2:**
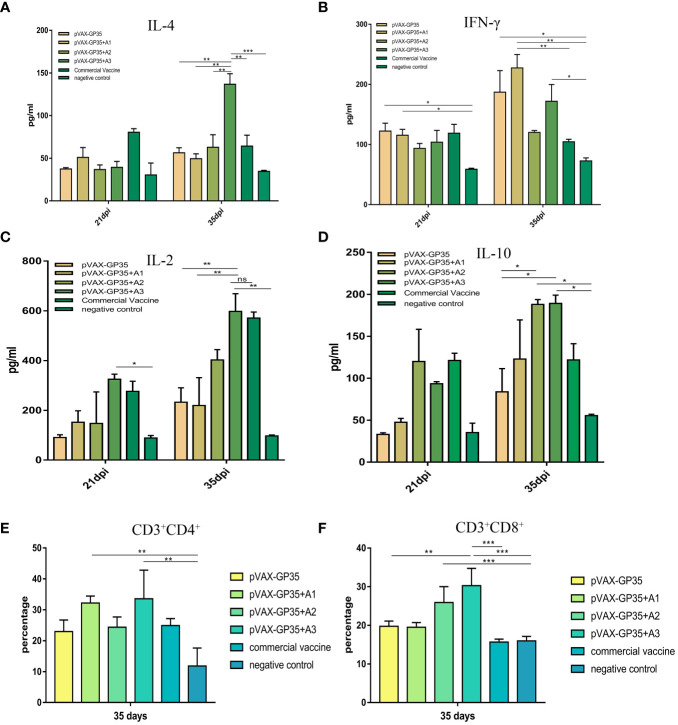
Detection of the level of cytokine secretion in the serum and CD3+CD8+ and CD3+CD4+ T-cell subtype from all groups. **(A)** The mean concentrations (pg/ml) of IL-4, as well as **(B)** IFN-γ **(C)** IL-2, and **(D)** IL-10 in the serum. **(E)** The percentage of CD3+CD4+ T-lymphocyte subpopulations in PBMC. **(F)** The percentage of CD3+CD8+ T-lymphocyte subpopulations in the PBMC. Data were presented as the mean ± S.E of 3 data in each group. (*P < 0.05, **P < 0.01, ***P < 0.001). ns, no significant differences.

At 21 and 35 dpi, serum IL-4 in all groups were detected (**Figure 2A**). At 35 dpi, all vaccine groups outperformed the NC group and the pVAX-GP35+A2 group was comparable to the commercially available inactivated vaccine group. The maximum concentration of IL-4 was 136.77 pg/mL in the pVAX-GP35+A3 group, 2.4 times greater than that of the pVAX-GP35 group (P<0.01) and 2.13 times greater than that of the commercial inactivated vaccine group (P<0.01). At 21 and 35 dpi, IFN-γ was detected (**Figure 2B**). The IFN-γ concentrations measured in the pVAX-GP35, pVAX-GP35+A1, and pVAX-GP35+A3 groups were greater than that of the NC group at 21 dpi (P<0.05). At 35 dpi, the IFN-γ concentration in the pVAX-GP35+A1 group was 227.37 pg/mL, which was higher than that of the NC group (P<0.01) and 2.17 times greater than that of the commercial inactivated vaccine group (P<0.05).

Serum IL-2 was evaluated at 21 and 35 dpi (**Figure 2C**). At both 21 (P<0.05) and 35 dpi (P<0.01), the pVAX-GP35+A3 group outperformed the NC group. The group also outperformed the pVAX-GP35 (P<0.01) and pVAX-GP35+A1 (P<0.01) groups. For IL-10 (**Figure 2D**), the pVAX-GP35+A2 and pVAX-GP35+A3 groups outperformed the pVAX-GP35 group (P<0.05) and NC group (P<0.05) at 35 dpi.

The CD3^+^CD4^+^ and CD3^+^CD8^+^ T cell subsets of the pigs’ peripheral blood lymphocytes were examined at 35 dpi. **Figure 2E** depicts the results of the CD4^+^ T lymphocyte subset analysis. The pVAX-GP35+A1 group had a greater percentage of CD4^+^ T cells (32.2%) than the NC group (P<0.01), and was found to be somewhat higher than measured for the commercial vaccine (P>0.05) and pVAX-GP35 groups (P>0.05). Analysis of the blood from the pVAX-GP35+A3 group indicated that 33.56% of the T lymphocytes were CD4^+^ T cells, which was higher than that of the NC group (P<0.01). The group also outperformed the commercially available inactivated vaccine (P>0.05) and pVAX-GP35 groups (P>0.05). **Figure 2F** depicts the results of CD8^+^ T lymphocyte subset analysis. The blood from the pVAX-GP35+A3 group was shown to contain a higher percentage of CD8^+^ T lymphocytes (30.27%) compared to the NC (P<0.001) and commercial vaccine groups (P<0.001); this was also higher than observed for the pVAX-GP35 group (P<0.01). The percentage of CD8^+^ T lymphocytes in the pVAX-GP35+A2 group was greater than that of the NC group (P<0.001).

### Pig health status, viral loads and histopathology after PRRSV challenge

**Figure 3:**
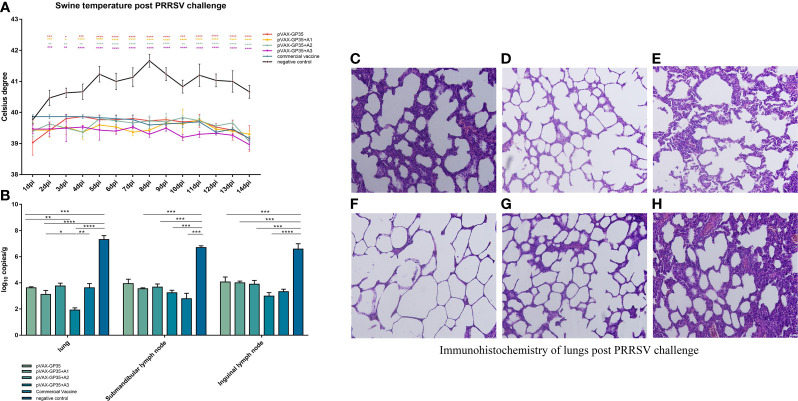
Assessment of protective effect of the recombinant candidate vaccines after challenge. **(A)** Pigs' temperatures were monitored daily after being challenged by PRRSV. None of the immunization groups exceeded 40 degrees Celsius. **(B)** Viral loads in pig tissues were challenged by the PRRSV. **(C–H)** Pathological sections of lungs post PRRSV challenge. Moderate interstitial pneumonia was observed in vaccined pigs with consolidation. Interstitial pneumonia was observed and severe alveolar septum thickening in negative group pigs. **(C)** lung of pVAX-GP35 group. **(D)** lung of pVAX-GP35+A1 group. **(E)** lung of pVAX-GP35+A2 group. **(F)** lung of pVAX-GP35+A3 group. **(G)** lung of commercial vaccine group. **(H)** lung of NC group. All images magnification of 200×. (Data are presented the mean ± S.E of 3 data. *P < 0.05, **P < 0.01, ***P < 0.001, ****P < 0.0001). For the line graph, the colored symbols represent the immunization group compared to the commercial vaccine.

Pig rectal temperatures were measured daily after being challenged by PRRSV (**Figure 3A**). The rectal temperatures of the pVAX-GP35, pVAX-GP35+A1, pVAX-GP35+A2, and pVAX-GP35+A3 groups did not exceed 40.0 °C. The rectal temperatures recorded for the commercial vaccine group were lower than 40.0 °C but higher than those recorded for the pVAX-GP35+A1 and pVAX-GP35+A3 groups. The temperatures recorded for the NC group rose above 40.0°C two days after the challenge and never dropped below 40.5°C within a 14-day period, reaching a maximum of 41.7°C at 8 dpi.

The Activities of Daily Living assessment measured the pigs’ health status after the PRRSV challenge (**Table 1**). Following PRRSV challenge, the NC group displayed little or no feeding activity, preferred to cluster and crawl together and exhibited coughing and flushed skin. The pigs in the pVAX-GP35+A3 group ate normally, breathed normally, and had regular exercise intention.

**Table 1:** ADL assessment for pigs after 14 dpi by PRRSV challenge.

	pVAX-GP35	pVAX-GP35+A1	pVAX-GP35+A2	pVAX-GP35+A3	Commercial vaccine	Negative control
Appetite	2	2	2	3	3	0
Mental state	1	2	1	2	2	1
Skin color of head	2	2	2	2	2	2
Breathing	2	2	2	2	2	1
Over all	7	8	7	9	9	4

On a scale of 0-3, 0 represents severe symptoms, 1 represents moderate symptoms, 2 represents mild symptoms, and 3 represents no obvious symptoms.

We harvested the lungs, submandibular lymph nodes, and inguinal lymph nodes from the pigs of all groups 14 days post-PRRSV challenge, and the virus titers were evaluated by qPCR. The results demonstrated that the immunized groups had lower viral loads in their primary organs compared to the NC group (**Figure 3B**). The pVAX-GP35+A3 group had the lowest viral load compared to the NC group (P<0.001). This group also exhibited lower viral loads in the lung and inguinal lymph node specimens compared to the commercial vaccine group. Furthermore, the pVAX-GP35+A1 group had lower viral loads in the lung compared to the commercial vaccine group. As a result, these findings suggest that recombinant DNA vaccines can lower the viral loads of inoculated pigs following a PRRSV challenge.

At 14 days after the virus challenge, pathological sections of the lungs were assessed (**Figures 3C–H**). After the challenge, the lungs of the pigs in the pVAX-GP35+A3 group were normal and better than in the pVAX-GP35 group. Conversely, in the NC group, lung interstitial widening, alveolar wall hyperplasia, and significant inflammatory cell infiltration were observed.

## Discussion

PRRSV can induce miscarriage and respiratory illness and make pigs more susceptible to other swine viruses. Vaccination can provide good protection against PRRSV infections in pigs; however, MLV and inactived vaccines will not achieve the anticipated goals since they are associated with several limitations, including an inability to confer sterilizing immunity (Renukaradhya et al., 2015). DNA vaccines have the potential to stimulate humoral and cellular immunity against PRRSV challenge, potentially saving the pig industry millions of dollars (Hobernik and Bros, 2018).

Plasmid DNA is relatively stable at ambient temperature, eliminating the requirement for a cold chain during transport (Sharma et al., 2020). The production of plasmids for use in DNA vaccines eliminates the need for infectious pathogen protein purification, enhancing their safety profile further. In addition, DNA vaccines have an outstanding safety record in the clinic (Li and Petrovsky, 2016). One of the most significant concerns concerning DNA vaccines is the likelihood of transfected DNA becoming integrated into the genomes of somatic and germ cells, which results in an imbalance in gene expression and mutation (Hobernik and Bros, 2018). However, Wang and colleagues calculated that the integration frequency was far lower than the number of spontaneous gene alterations (Wang et al., 2004). Another study indicated that the bulk of the plasmid DNA injected into the skeletal muscles of mice remained at the injection site, with tiny percentages detected in other organs, including the gonads, but not incorporated into the genome (Manam et al., 2000). Long-term reporter expression was achieved in primates after repeated intramuscular injections of a luciferase-encoding reporter vector, but no anti-DNA antibodies were generated (Jiao et al., 1992). DNA vaccines have been effective in recent human studies (Kim et al., 2014; van Diepen et al., 2019; Tebas et al., 2021), including against Ebola, influenza H5N1, influenza H1N1, and Zika, where phase I or phase II clinical trials were conducted (Rauch et al., 2018).

The immunostimulatory qualities of the adjuvant chosen in combination with the specific antigen determine the efficiency of DNA vaccines (Leroux-Roels, 2010). Based on its capacity to activate an innate immune response, defined by the activation of professional APC and B-cells, CpG ODN was considered a suitable vaccination adjuvant (Krieg et al., 1995; Klinman et al., 1996). No detrimental health effects have been documented in trials involving the use of CpG ODN in nonhuman primates (Klinman et al., 1997; Verthelyi et al., 2002). In the present study, two adjuvants were evaluated. The A1 adjuvant was a saponin extract. Saponin consists of a hydrophobic core with a triterpenoid structure and a tension-active glycoside attached to the carbohydrate chain (Reichert et al., 2019). Saponin can affects the immune system and nervous system activities in animals (Reichert et al., 2019). The A2 adjuvant was a continuous aqueous emulsion water-in-oil-in-water adjuvant. Biphasic emulsions can deliver both lipophilic compounds and easily release water-soluble compounds (Sawant et al., 2021). Significantly, they can trigger both immediate and long-term immunological responses. Through the oil film, the compound emulsion can wrap some active components dissolved in water and release them slowly and steadily into the external aqueous phase (Benichou et al., 2004). The A3 adjuvant evaluated in this study was a combination of adjuvants A1 and A2.

PRRSV GP3 and GP5 were chosen as antigens in this investigation. A Kozak sequence was introduced in front of the protein sequence to increase target protein expression, and CpG sequences were put downstream the protein sequence to improve the immunological effect. pVAX-GP35+A3 activated the highest levels of neutralizing antibodies *in vivo*, with a titer of 1:19. The neutralizing antibody titers of pVAX-GP35+A1, pVAX-GP35+A2, and pVAX-GP35+A3 were more significant at 35 dpi than those elicited by the DNA vaccines pIR-VR2385-CA-dORF2 (Pujhari et al., 2013). Similarly, the vaccines in this study were higher than those elicited by the DNA vaccines pEGFP-IL18-GP5 and pEGFP-GP5 (Zhang et al., 2013). Likewise, the vaccines in this study shown better neutralizing antibody titers than the DNA vaccines pcDNA3.1-SynORF5, pcDNA3.1-PoIFN-1-SynORF5, and BPEI/PLGA-SynORF5, which elicited neutralizing antibody titers of 1:8, 1:12, and 1:14, respectively (Du et al., 2017). In this study, commercial inactived vaccine group showed higher neutralizing antibodies than pVAX-GP35+A3 group, but viral load and pathology section results found limited protection, which provided weaker protection against PRRSV challenge than the pVAX-GP35+A3 group. These results suggest that not only is antibody-dependent humoral immunity against PRRSV attack, but that cellular immunity is also key to providing protection.

pVAX-GP35+A3 has the potential to boost cellular immune responses in inoculated pigs by increasing CD4^+^ and CD8^+^ T-cell numbers. GP3 and GP5 can increase not only humoral immunity, but also cellular immunity against PRRSV, according to the results. PRRSV viral load measurements were employed for the challenge protection experiment at 35 dpi to confirm the immunological effect in the experimental groups. The viral loads in the lung tissues harvested from the pVAX-GP35+A3 group were lower than those observed following vaccination with the DNA vaccines pcDNA3.1-SynORF5, pcDNA3.1-PoIFN-1-SynORF5, and BPEI/PLGA-SynORF5 (Calvet et al., 2014). Given these results, pVAX-GP35+A3 may provide more effective protection in pigs. Although the virus was still detectable in the tissues of pigs at the end of the experiment, viral loads in the lungs, submandibular lymph-nodes and inguinal lymph-nodes from pigs in the pVAX-GP35+A3 group were significantly lower than in the same tissues harvested from the NC group. Evidence indicates that pVAX-GP35+A3 can successfully restrict viral replication in pigs.

However, PRRSV can be detected in pigs immunized with the DNA vaccine and the efficacy of DNA vaccines requires further improvement. The mode of delivery should also be modified to boost the immunological response. Different vaccine delivery methods, including soluble microneedle patches, surface electroporation, and intradermal inoculation with needles are all effective DNA delivery methods in pigs (Bernelin-Cottet et al., 2019). Fractional non-ablative laser treatment may also improve DNA immunogenicity (Wang et al., 2015). Applicators have coated microneedle arrays that are spring-loaded (Fernando et al., 2018), and could minimally invasive tissue penetration and local retention. which can leading to continuous delivery of DNA vaccines, also prolong the protection time of the vaccine. DNA vaccines still need to be improved in terms of antigen selection, adjuvant matching, and delivery mechanisms.

## Data availability statement

The raw data supporting the conclusions of this article will be made available by the authors, without undue reservation.

## Ethics statement

The animal study was reviewed and approved by IACUC of AMMS- 11 - 2021 - 012.

## Author contributions

GZ, HL, and NJ conceived the study. GZ, WS and CX contributed to data collection, analysis. SW contributed to interpretation. JZ, HZ, YG, XC and FN helped with data visualization. GZ, YiZ and YaZ completed the drafting of the manuscript. JZ, XZ revised the manuscript. SF supervised the research. All authors contributed to the article and approved the submitted version.

## Funding

This work was supported by National Program on Key Research Project of China [2018YFD0500104] and [2018YFD0500803], National High Technology Research and Development Program of China [2011AA10A208], and [CAAS-ASTIP-2017-ISAPS].

## Conflict of interest

The authors state that there were no commercial or financial relationships that may be considered as a potential conflict of interest during the research.

## Publisher’s note

All claims expressed in this article are solely those of the authors and do not necessarily represent those of their affiliated organizations, or those of the publisher, the editors and the reviewers. Any product that may be evaluated in this article, or claim that may be made by its manufacturer, is not guaranteed or endorsed by the publisher.
